# Single‐cell RNA sequencing reveals new subtypes of lens superficial tissue in humans

**DOI:** 10.1111/cpr.13477

**Published:** 2023-04-14

**Authors:** Meng‐Chao Zhu, Wei Hu, Lei Lin, Qing‐Wen Yang, Lu Zhang, Jia‐Lin Xu, Yi‐Tong Xu, Jia‐Sheng Liu, Meng‐Di Zhang, Xiao‐Yu Tong, Kai‐Yi Zhu, Ke Feng, Yi Feng, Jian‐Zhong Su, Xiu‐Feng Huang, Jin Li

**Affiliations:** ^1^ State Key Laboratory of Ophthalmology, Optometry and Visual Science, Eye Hospital Wenzhou Medical University Wenzhou China; ^2^ National Clinical Research Center for Ocular Diseases, Eye Hospital Wenzhou Medical University Wenzhou China; ^3^ Department of Integrative Medicine and Neurobiology, School of Basic Medical Sciences, Institutes of Brain Science, Brain Science Collaborative Innovation Center, State Key Laboratory of Medical Neurobiology, Institute of Acupuncture and Moxibustion, Fudan Institutes of Integrative Medicine Fudan University Shanghai China; ^4^ Zhejiang Provincial Clinical Research Center for Pediatric Disease The Second Affiliated Hospital of Wenzhou Medical University Wenzhou Zhejiang China

## Abstract

Although the cell atlas of the human ocular anterior segment of the human eye was revealed by single‐nucleus RNA sequencing, whether subtypes of lens stem/progenitor cells exist among epithelial cells and the molecular characteristics of cell differentiation of the human lens remain unclear. Single‐cell RNA sequencing is a powerful tool to analyse the heterogeneity of tissues at the single cell level, leading to a better understanding of the processes of cell differentiation. By profiling 18,596 cells in human lens superficial tissue through single‐cell sequencing, we identified two subtypes of lens epithelial cells that specifically expressed C8orf4 and ADAMTSL4 with distinct spatial localization, a new type of fibre cells located directly adjacent to the epithelium, and a subpopulation of ADAMTSL4^+^ cells that might be lens epithelial stem/progenitor cells. We also found two trajectories of lens epithelial cell differentiation and changes of some important genes during differentiation.

## Graphical abstract

scRNA‐seq reveals three cell types in lens superficial tissue with two differentiation trajectories between them, and a cluster might be lens epithelial stem/progenitor cell.

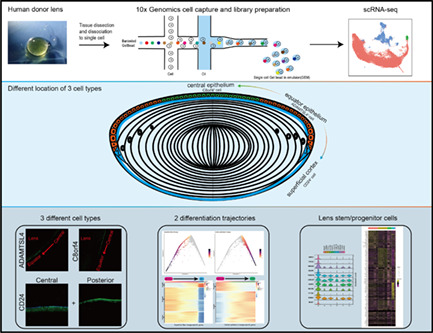

## 1 INTRODUCTION

The human crystalline lens, which is a transparent and avascular biconvex tissue, is an important structure in the refractive system of the eye to regulate light focusing.^1^ It is enveloped by a basement membrane known as the capsule.^2^ The lens consists of a layer of lens epithelial cells (LECs) under the anterior capsule and lens fibre cells (LFCs), which form most of the lens.

LECs proliferate and differentiate into LFCs throughout life,^3^, ^4^, ^5^ and LECs have a different cellular morphology from anterior of the lens to the equator. A study of the mouse lens found that LECs at the equatorial region express genes that are not expressed in the anterior of the lens.^6^ However, most studies are conducted in animal models as well as cell lines in vitro. The cell atlas of the human ocular anterior segment was revealed by single‐nucleus RNA sequencing (snRNA‐seq) of isolated nuclei from frozen tissue samples.^7^ However, single‐cell RNA sequencing (scRNA‐seq) of intact single cells of fresh lens samples has not been reported. Additionally, whether subtypes of lens stem/progenitor cells exist among epithelial cells and the molecular characteristics of cell differentiation of the human lens remain unclear.

scRNA‐seq has evolved in recent years and become an important tool to study cellular heterogeneity and various physiological processes because of its single cell level of resolution, high sequencing throughput and depth, and availability of analytical tools.^8^, ^9^, ^10^ scRNA‐seq has a higher gene detection rate and detection sensitivity than snRNA‐seq, and its specifically enriched genes are ribosomal and mitochondrial, whereas snRNA‐seq loses transcripts that mainly localize in the cytoplasm because of its input material is nuclear RNA rather than cellular RNA.^11^

By harnessing single‐cell transcriptomics of superficial tissues of human lens, including LECs and early fibre cells, we found that two subtypes of LECs located in different regions express different markers and a subpopulation of ADAMTSL4^+^ cells that specifically express stem cell‐related genes, which might be lens epithelial stem/progenitor cells. We also identified a new type of young LFC in the lens superficial tissue. We aimed to understand the molecular characteristics of cell differentiation in superficial tissue of the human lens and identified changes in expression of some important genes in the process of lens cell differentiation.

## 2 MATERIALS AND METHODS

### 2.1 Tissue processing

Eleven donated lenses of 10 donors, of which D2 provided two lenses, without a history of ocular disease were obtained from the Eye Bank Center of Wenzhou Medical University (Table 1). Donated eyes were obtained post‐mortem within <6 h from death and dissected within 1 h of enucleation. Four lenses were used for scRNA‐seq and the additional six lenses were used for immunofluorescence and tissue clearance (Table 1).

**TABLE 1:** Donor information.

Sample	Sex	Age	Sample	Method
D1	Male	5 years	Whole len's superficial cells	scRNA‐seq
D2	Male	15 years	Whole len's superficial cells	scRNA‐seq
D3	Male	79 years	Whole len's superficial cells	scRNA‐seq
D4	Male	55 years	Len's superficial cells without anterior central 6 mm zone	scRNA‐seq
D5	Male	15 years	Lens sagittal sections	Immunofluorescence
D6	Male	49 years	Lens sagittal sections	Immunofluorescence
D7	Female	69 years	Lens sagittal sections	Immunofluorescence
D8	Female	6 months	Whole lens	Tissue clearance
D9	Male	17 years	Whole lens	Tissue clearance
D10	Male	49 years	Whole lens	Tissue clearance

Abbreviation: scRNA‐seq, single‐cell RNA sequencing.

For samples D1–3, the entire lens capsule (less the posterior 4 mm capsulorhexis) containing all lens epithelia and early fibre cells was used. A circular capsulorhexis of the central 4 mm of the posterior capsule was performed, and the remaining lens capsule with superficial lens cells was sheared radically from the posterior margin to the equator using surgical scissors. After removing fibre cells from the nuclear portion of the lens, the fibre cells in the cortical portion of the lens were carefully removed. D4 was treated similarly to D1–D3, except the most central anterior cells (6 mm capsulorhexis) were removed. We collected the capsule without the central 6 mm anterior zone by a circular central anterior capsulorhexis, and the remaining capsule with superficial lens cells was collected by the same procedure described above.

This study was approved by the Laboratory Human Ethics Committee of Wenzhou Medical University (2020‐132‐K‐117‐02) and was carried out in compliance with the Declaration of Helsinki.

### 2.2 scRNA‐seq

Lens superficial cells were digested in 600 μL digestion solution consisting of 400 μL pancreatin (0.25%), 100 μL collagenase A (2 mg/mL) and 100 μL dispase II (10 mg/mL) for 8 min at 37°C and gently pipetted 20 times to assist cell dissociation. Then, 600 μL DMEM containing 10% FBS was added to the digestion solution for neutralization. The capsule was removed after neutralization, and the sample was passed through a 40‐μm filter. The obtained single‐cell suspension was centrifuged at 200*g* for 5 min, the supernatant was discarded, and the cells were resuspended in 1150 μL phosphate‐buffered saline (PBS). The single‐cell suspension (300–600 live cells/μL determined by Count Star) was immediately used for scRNA‐seq with 10x Genomics.

### 2.3 Bioinformatics analysis of scRNA‐seq data

Reads were aligned and unique molecular identifier counts were obtained using CellRanger pipeline (10x Genomics). For further processing, integration and downstream analysis, R (version 4.1.1) and Seurat (version 4.1.0) were used.^12^ Cells with gene numbers of <500 and >7500, and potential stress signals of >20% mitochondrial reads were excluded. The sequencing depth was normalized for each cell in the dataset, and Seurat‐based canonical correlation analysis was performed on D1–3 sample datasets to remove inter‐sample batch effects. The dataset was subsequently subjected to linear dimensionality reduction by principal component analysis (PCA). PCA dimensions were evaluated and selected by the results of elbow plots. Data were clustered in R using a Louvain graph algorithm. The cluster resolution was selected by the results of the clustree (version 0.4.4) program.^13^ Cells were projected onto two‐dimensional coordinates using the umap algorithm. Marker genes for lens epithelium and fibre cells were used to identify cell types. Epithelial clusters were extracted for reclustering, and differentially expressed genes between clusters were identified using the FindAllMarkers function. Gene ontology (GO) functional enrichment analysis was performed using the clusterProfiler package (version 4.2.2). Integrated analysis of intact capsule samples (D1–3) with ring retrieved capsule samples (D4) was performed using Seurat (version 4.1.0). To infer the differentiation trajectory and genes whose expression was significantly associated with the pseudotime, we used the destiny package (version 3.8.1).

### 2.4 Immunofluorescence

Three donated lenses were immediately fixed in a fixation solution (40% formaldehyde:absolute ethanol:double distilled water:glacial acetic acid = 1:4:4:1) for 48 h at 4°C, embedded in paraffin, and sectioned. Before staining, the tissue sections were dewaxed in xylene, rehydrated using a graded series of ethanol solutions and washed in distilled water. Then, the sections were placed in 100× citrate antigen retrieval solution in a 98°C water bath for 20 min. After cooling slowly to room temperature, the sections were washed with PBS three times, each time for 5 min, and blocked in 5% sheep serum and 1% bovine serum albumin in PBS for 1 h, and then incubated with primary antibodies against human CD24 (1:200, Cat. # ab202073, Abcam, Cambridge, MA, USA), ADAMTSL4 (1:100, Cat. # A4785, ABclonal, Wuhan, Hubei Province, China), and C8orf4 (1:100, Cat. # ab229680, Abcam) at 4°C overnight. An Alexa Fluor 488‐conjugated secondary antibody (1:800, Cat. # ab150077, Abcam) was applied and DAPI (Cat. # 62248, Thermo Fisher Scientific, Waltham, MA, USA) was used for counterstaining. Images were obtained under a Zeiss LSM710 confocal microscope.

### 2.5 iDISCO tissue clearing, 3D imaging and Imaris analysis

The iDISCO process was based on the protocol of Renier et al.^14^ A fresh lens was fixed with paraformaldehyde for 48 h before the clearing process. Then, a gradient of methanol (20%, 40%, 60%, 80% and 100% methanol/PBS for 1 h each at room temperature) for dehydration and incubation in 66% dichloromethane/33% methanol with shaking were applied. Because of a colour change caused by fixation in paraformaldehyde, the lens was bleached with H_2_O_2_ (5% H_2_O_2_/95% methanol, shaking at 4°C overnight), which was followed by rehydration in gradient methanol (80%, 60%, 40% and 20% methanol/PBS for 1 h each at room temperature). Immunostaining employed traditional immunofluorescence methods, including permeabilization and blocking, primary and secondary antibody incubation (CD24, Cat. # EPR19925, Abcam; donkey anti‐rabbit secondary antibody, Alexa Fluor™ 594, Cat. # A‐21207, Thermo Fisher Scientific; DAPI). Then, dehydration in methanol and incubation in dichloromethane were repeated as described above. Dibenzyl ether was used to calibrate reflection index for approximately 48 h.

A Lightsheet microscope (Nuohai Life Science Co., Ltd) was used for 3D imaging with Imaris software (Imaris 9.7, Oxford Instruments PLC, UK) for quantitative analysis. Surface algorithms were used for remodelling and thickness calculation.

## 3 RESULTS

### 3.1 Different cell types exist in the superficial tissue of lens

**FIGURE 1:**
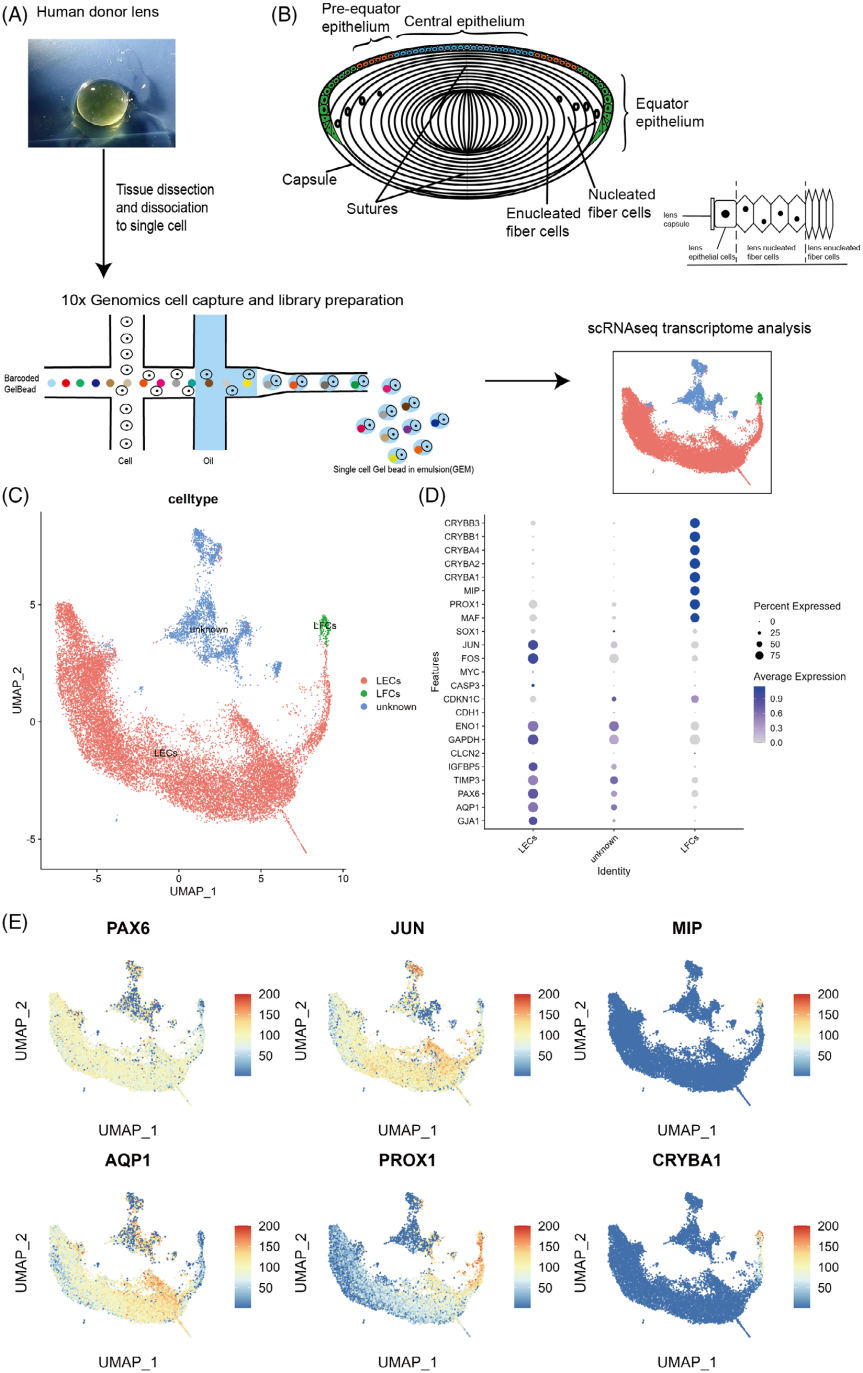
Single‐cell RNA sequencing (scRNA‐seq) identified three clusters of lens superficial tissue. (A) Scheme for scRNA‐seq. (B) Schematic diagram of the adult lens. (C) Uniform manifold approximation and projection (UMAP) of 21,711 single cells with three clusters, lens epithelial cells (LECs), lens fiber cells (LFCs), and unknown. (D) Dot plot of the cluster‐defining genes of lens epithelial and fibre cells. (E) PAX6 and AQP1 were specific to LECs, JUN and PROX1 were related to epithelial differentiation, and MIP and CRYBA1 were specific to LFCs.

Lens epithelial and superficial fibre cells from four donor lenses of various ages were used for single‐cell RNA sequencing. After filtering low‐quality cells, the transcriptome profiles of 21,711 cells were further analysed (D1: 10373 cells; D2: 5135 cells; D3: 6203 cells) (Figure 1A).

Data of 21,711 single cells were embedded in a uniform manifold approximation and projection (UMAP). Using unbiased low‐resolution clustering, they were divided into three major cell clusters using established lens markers and annotated as epithelial and fibre cells (Figure 1C–E).^15^ The unknown cluster was relatively isolated from the other two clusters in the UMAP plot (Figure 1C). After integration to remove inter‐sample batch effects, the different samples did not overlap in this cluster (Figure S1A). Moreover, the expression of lens markers was relatively weak compared with the other two clusters (Figure 1D). Additionally, the gene expression profiles were similar to that of the lens epithelial cluster (Data S1). Therefore, this cluster may have contained different subpopulations reflecting the heterogeneity of different ages, considering that the biological replicates in this study were insufficient for age difference analysis and the unknown cluster was not analysed further.

### 3.2 Identification of two subpopulations of LECs and one LFC cluster with novel specific markers by scRNA‐seq

**FIGURE 2:**
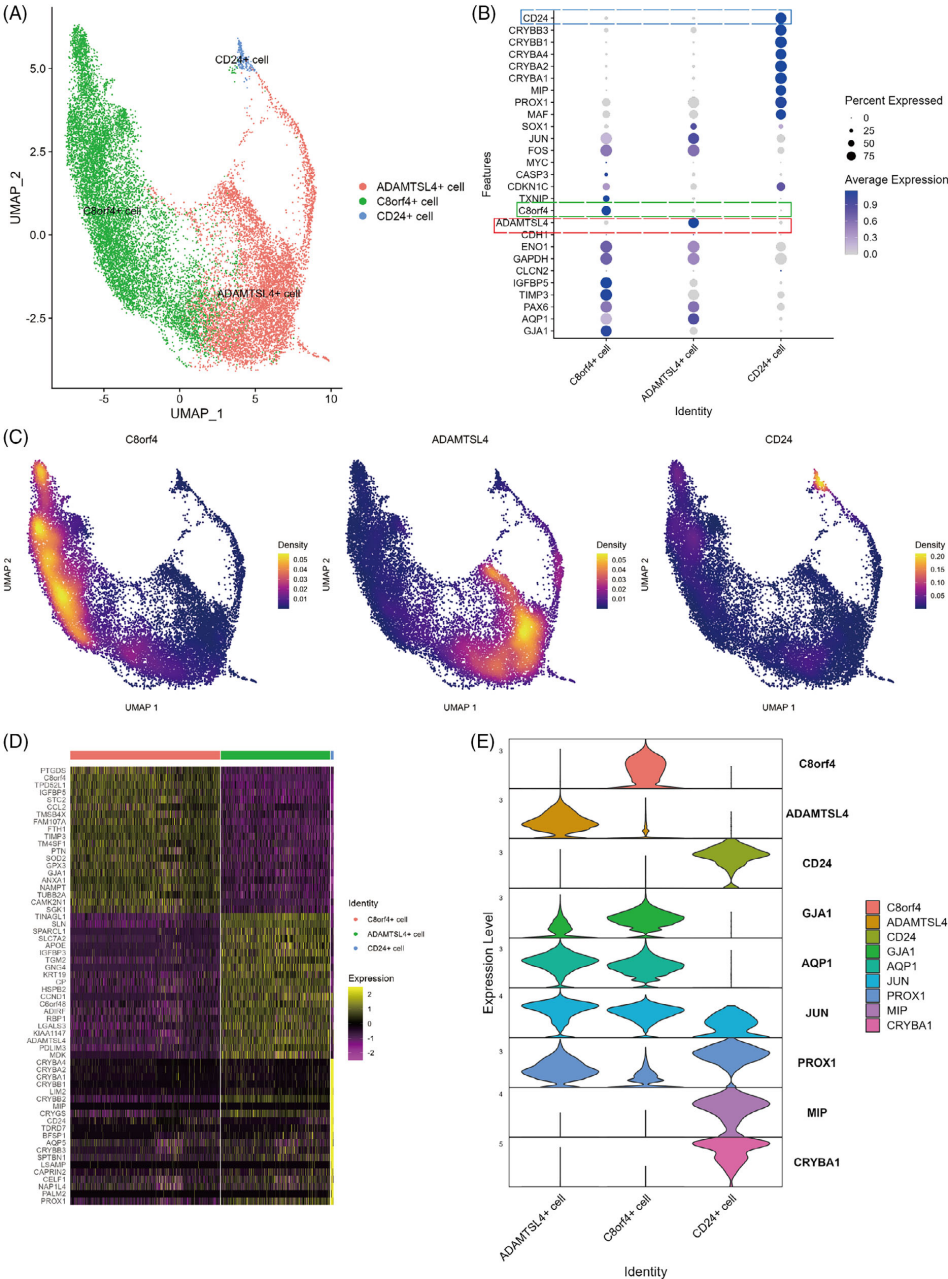
Three types of lens epithelial cells and fibre cells. (A) uniform manifold approximation and projection (UMAP) of epithelial and fibre cells after reclustering. (B) Dot plot of the cluster‐defining genes. (C) UMAP of C8orf4, ADAMTSL4 and CD24 expression indicated by kernel gene‐weighted density estimation. (D) Heat map of the top 20 differentially expressed genes for each cluster revealed distinct transcriptomic profiles of the three cell clusters and allowed cell cluster identification. (E) Expression of cluster marker genes (C8orf4, ADAMTSL4 and CD24), lens epithelial cell‐specific genes (GJA1 and AQP1), differentiation‐related genes (JUN and PROX1), and lens fibre cell‐specific genes (MIP and CRYBA1) in the three clusters.

Data of 18,596 sequenced cells belonging to fibre and epithelial cell clusters were extracted for reclustering analysis (D1: 9970 cells; D2: 3686 cells; D3: 4940 cells). As shown in Figure 2A, we identified three clusters including one LFC cluster and two LEC clusters, in which the proportion of cells in the three samples was similar (Figure S1B). Expression of LEC markers was not identical in the two epithelial cell clusters. C8orf4 and ADAMTSL4 genes were specifically expressed in the two epithelial cell clusters, respectively (Figure 2B,C,E), and CD24 was specifically expressed in the LFC clusters (Figure 2B,C,E). The expression similarities suggested that C8orf4^+^ and ADAMTSL4^+^ cells were LECs, but differed in expression of specific markers. After performing differential analysis of gene expression among the three clusters (Figure 2D), we found that the three clusters were three cell types with different gene expression profiles.

### 3.3 Different cell types have different localization in lens superficial tissue

Differential gene expression analysis of the three clusters showed that C8orf4 and ADAMTSL4 were expressed at high levels in the two subpopulations that expressed classical LEC markers, whereas CD24 was highly expressed in the cluster expressing classical LFC markers.

**FIGURE 3:**
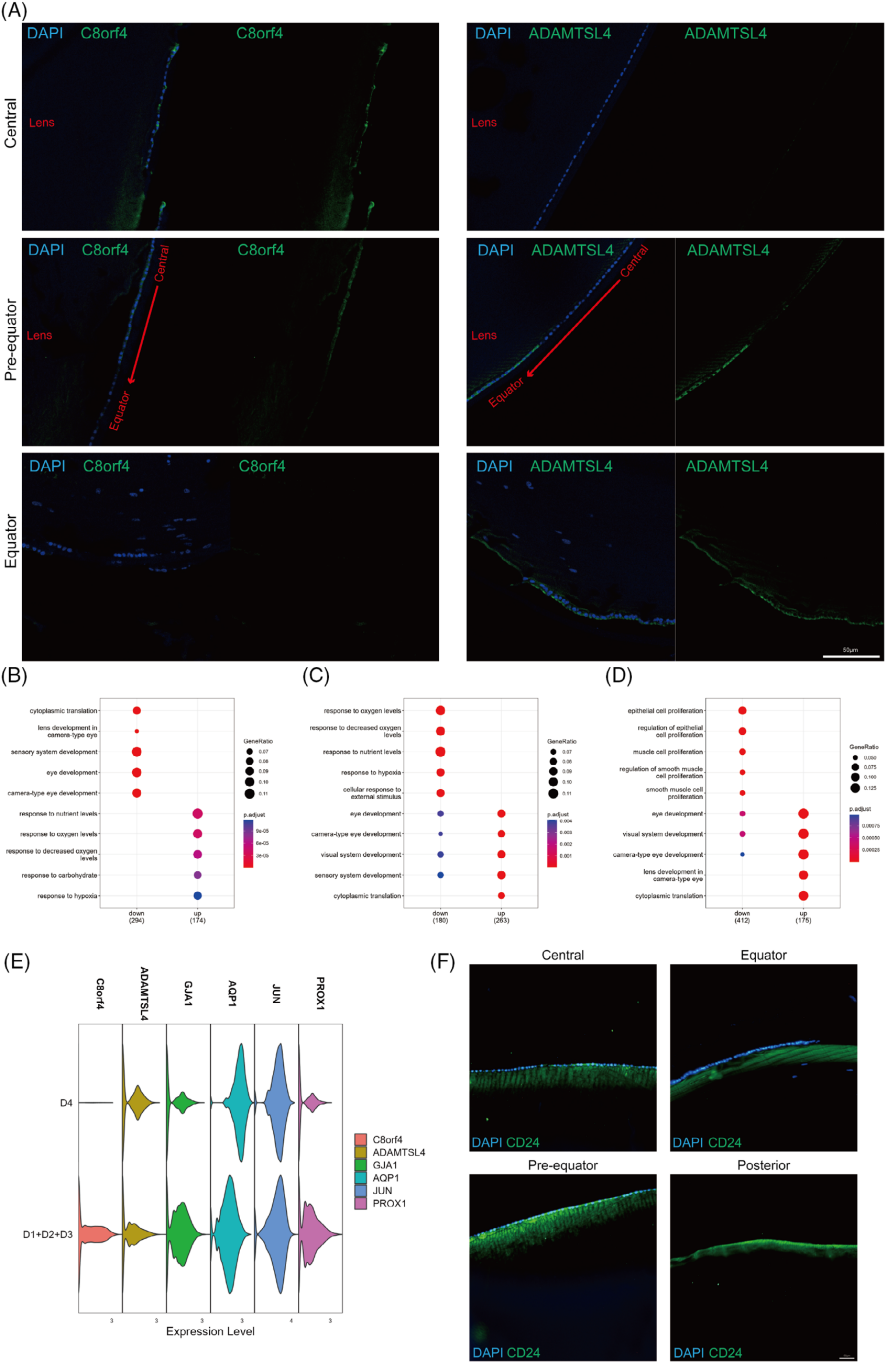
The three types of cells had different locations and functions. (A) Immunofluorescence of C8orf4 and ADAMTSL4 (green) in human lens tissue. GO analysis of intercluster gene expression comparing specific C8orf4^+^ cell (B), ADAMTSL4^+^ cell (C), and CD24^+^ cell (D) populations. (E) Violin plot of marker gene expression in D1/D2/D3 samples and the D4 sample. (F) Immunofluorescence of CD24 (green) in human lens tissue.

**FIGURE 4:**
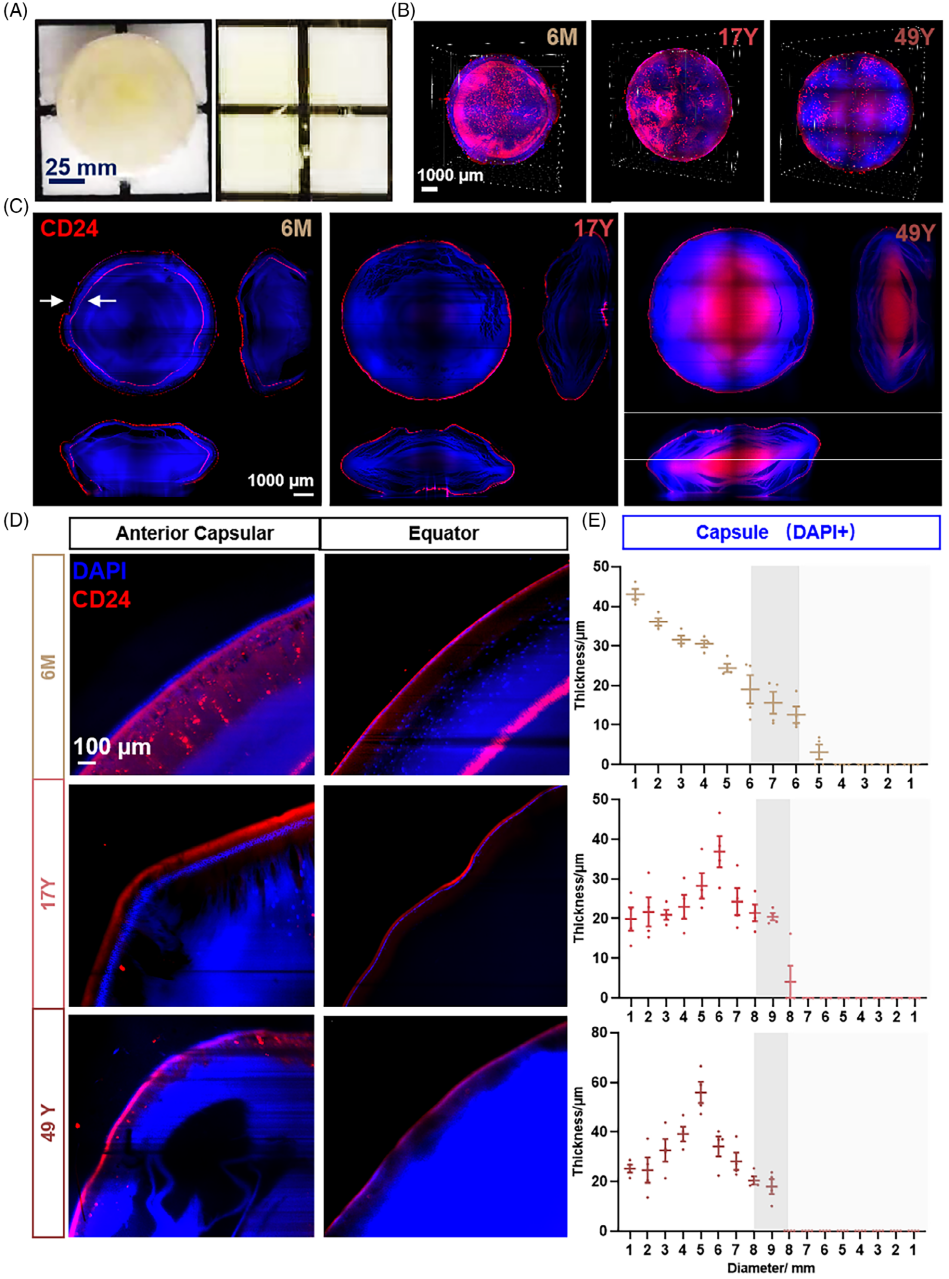
Tissue clearance staining of CD24. (A) Lens transparency after iDISCO processing. (B) Three‐dimensional view of CD24 (red) in the human lens of three different ages. (C) Axial section of CD24 (red) of the human lens of three different ages. (D, E) Spatial location and thickness of nuclei and CD24 from the anterior capsule to the equator and posterior capsule.

To confirm our findings, marker expression in the three clusters was assessed by immunofluorescence (Figure 3A). C8orf4 was mainly expressed in the anterior central region of the lens epithelium, and ADAMTSL4 was mainly expressed in the equator and pre‐equatorial region of the lens epithelium (Figure 3A). Interestingly, CD24 was expressed only in superficial fibre cells directly adjacent to the lens epithelium, including the lens anterior and posterior regions (Figures 3A,F and 4).

Functional enrichment analysis based on differentially expressed genes showed that C8orf4^+^ cells were related to the response to nutritional and oxygen levels (Figure 3B), while ADAMTSL4^+^ cells were related to eye development (Figure 3C). Moreover, CD24^+^ cells showed downregulation of genes related to epithelial cell proliferation and upregulation of genes related to eye development, including some LFC markers (Figure 3D).

Considering the different localization of the two clusters of LECs, the equator region of the capsule with superficial lens cells from one donor lens was used for scRNA‐seq (D4: 4651 cells). The size of the central zone of the lens epithelium lacks a unified definition and standard, and specific dimensions may vary on the basis of factors such as researchers, measurement methods and tools employed. In general, the diameter of the central zone of the lens epithelium is within the range of 5 mm or less.^16^, ^17^ We believe that the capsule without the central 6 mm anterior zone removed all the central lens epithelium. Then, the data were compared with those from whole capsule samples. The difference in the expression profile of LEC markers as well as C8orf4 and ADAMTSL4 between whole capsule samples and the equator capsule sample reconfirmed the distinct localization of the LEC subpopulations (Figure 3E).

Lens transparency confirmed the location of the CD24^+^ fibre cell cluster. A 3D view and axial section of the lens also showed an obvious difference between 6 months (6M) and 17 and 49 years (17Y and 49Y, respectively) (Figure 4B,C). CD24^+^ fibre cells appeared to be two rings in 6M, whereas only one ring was observed in 17Y and 49Y lens. From the anterior capsule to the equator and posterior capsule, the thickness of nuclei (DAPI^+^) and CD24, together with their spatial localization was recorded. CD24 was expressed beneath the capsular epithelium (Figure 4C–E). Detail imaging of CD24 in the 6M lens is shown in Figure S2A, which obviously appeared as two rings. The outer ring gradually thinned from the front to the back, while the inner ring gradually thickened just over the equator (Figure S2B). CD24 appeared as a single ring in 17Y and 49Y lenses similarly to the outer ring in the 6M lens (Figure S2C). The unique double ring of CD24 in the 6M lens indicated a role of CD24 in lens development. In particular, the unique double ring staining in the 6‐month lens required further sample confirmation.

### 3.4 Characteristics of the differentiation of human lens superficial cells

**FIGURE 5:**
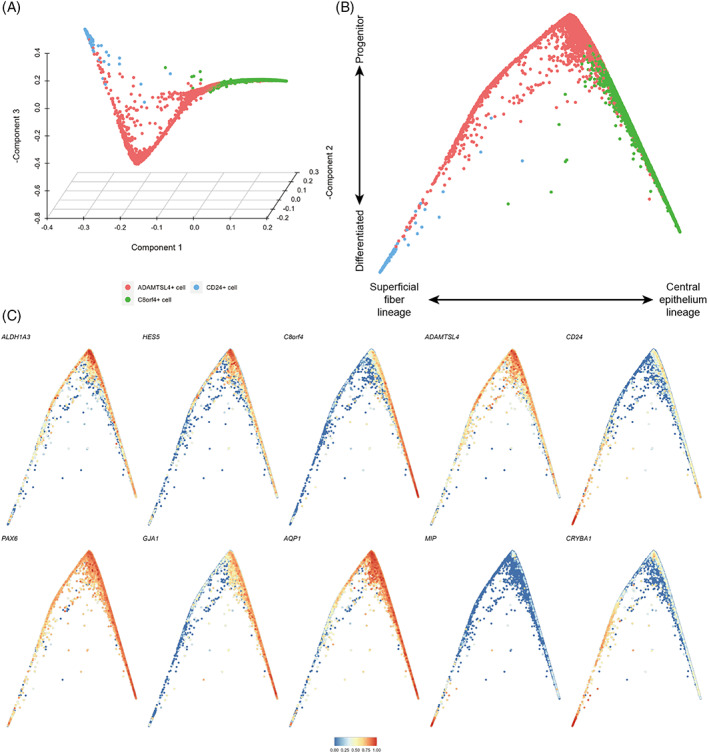
Computational reconstruction of differentiation processes in lens superficial tissue. (A) Diffusion map of lens superficial tissue, showing the first three diffusion components. (B) Differentiation trajectory of lens superficial tissue based on the first two diffusion components. (C) Same plot in (B) coloured by normalized and scaled expression values of various genes.

LECs differentiate into LFCs throughout life. However, the relationship between cell differentiation of the two epithelial cell subtypes and superficial fibre cells found in this study was unclear. We focused on these cells to investigate the differentiation status of lens superficial tissue. The differentiation states and transitions between them were computationally reconstructed using diffusion maps.^18^ This method embeds the data in a low‐dimensional space where distances between cells represent progression through a gradual but stochastic process such as differentiation. In the diffusion maps, the data showed a structure with gradual transitions between two different clusters and cells originating from a common origin (Figure 5A,B).

We found expression of progenitor cell markers ALDH1A3 and HES5,^19^ and ADAMTSL4^+^ cells gradually decreased as cells progressed away from their common origin (Figure 5C), which were derived from ADAMTSL4^+^ cells. We further noted that the left arm of the differentiation trajectory terminated at CD24^+^ cells and showed increasing expression of MIP, CRYBA1 and CD24 (Figure 5C), which was consistent with the superficial fibre cell phenotype. On the right arm of the differentiation trajectory, cells derived from ADAMTSL4^+^ cell transitioned to C8orf4^+^ cells, during which expression of GJA1, IGFBP5 and C8orf4 was increased, suggesting that this branch represented differentiation towards central epithelial cells (Figure 5C). In this branch, the expression levels of PAX6 and SOX2, which are considered to be markers of lens progenitor cells,^20^ remained relatively high (Figure 5C).

**FIGURE 6:**
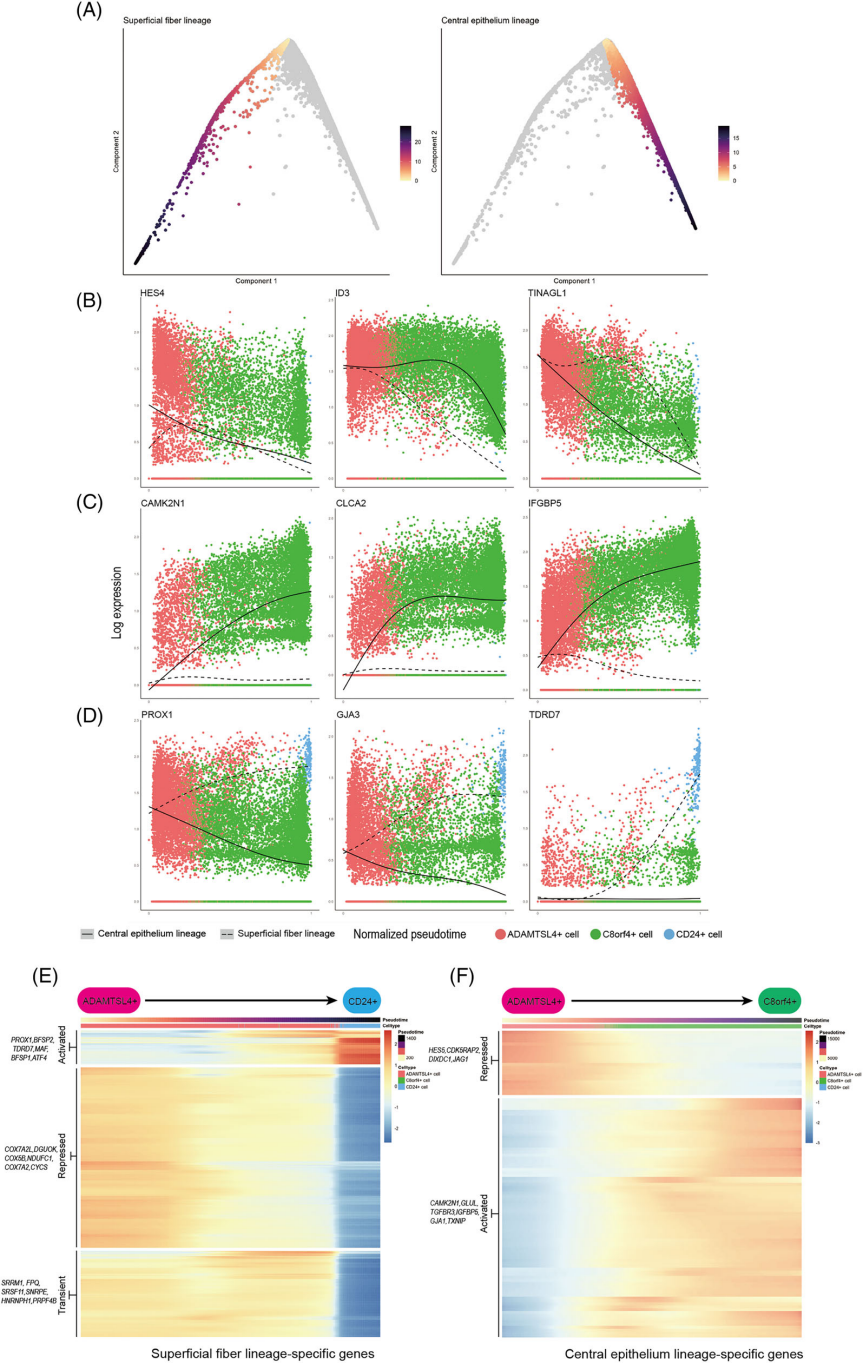
Pseudotime ordering identified genes associated with two trajectories. (A) Definition of the superficial fibre and central epithelium differentiation branch. Cells are coloured by their progression through pseudotime, where low values represent undifferentiated cells. (B–D) Examples of transcription factors with pseudotime‐dependent expression with the same overall trend in both branches (B) or branch‐specific trends (C, D). (E, F) Heat map of all genes with branch‐specific, pseudotime‐dependent expression for the superficial fibre lineage or central epithelium lineage (F). Values in the heat map are *z*‐scaled, spline‐smoothed expression values. Genes in heat maps were clustered by hierarchical clustering with a dynamic tree cut.

The diffusion map recapitulated superficial cells during the lens differentiation process, and therefore we computationally inferred with the two branches and ordered the cells in accordance with their progression through pseudotime (Figure 6A). This allowed us to identify genes with expression changes during the differentiation process. We found 71 genes that showed pseudotime‐dependent expression with the same directionality along both differentiation trajectories (Data S2). These included genes associated with known progenitor characteristics, such as HES4, ID3,^21^, ^22^ and FOXE3, which are associated with LEC differentiation,^23^, ^24^ and genes with no previously reported associated with lens differentiation, such as TINAGL1, PBX1 and SLC16A1 (Figure 5B). Additionally, we identified 905 genes with branch‐specific expression patterns. We then clustered the gene expression trend on each of the two branches and identified sets of genes that changed over pseudotime (Figure 6E,F, Data S3). On the superficial fibre cell branch, we found two clusters of genes with increased or decreased expression during differentiation. The increased cluster was enriched for genes involved in eye development and the LFC differentiation process (e.g., PROX1, BFSP2 and TDRD7, Figure 6E, Figure S3A). The decreased cluster was enriched for genes involved in energy metabolism (e.g., COX7A2L, PHGDH and DGUOK, Figure 6E, Figure S3B). Genes involved in RNA splicing showed a transient phase of upregulation during the superficial fibre cell differentiation process (e.g., SRRM1, SFPQ and SRSF11, Figure 6E, Figure S2C). During the differentiation process towards central epithelial cells, we identified two broad clusters of genes that gradually increased or decreased their expression. Genes with increasing expression were involved in the response to nutrient and oxygen levels and the epithelial cell proliferation process (e.g., CAMK2N1, GLUL and IGFBP5, Figure 6F, Figure S3D), while genes with decreasing expression were involved in negative regulation of cell differentiation and stem cell population maintenance (e.g., HES5, CDK5RAP2 and DIXDC1, Figure 6F, Figure S3E).

**FIGURE 7:**
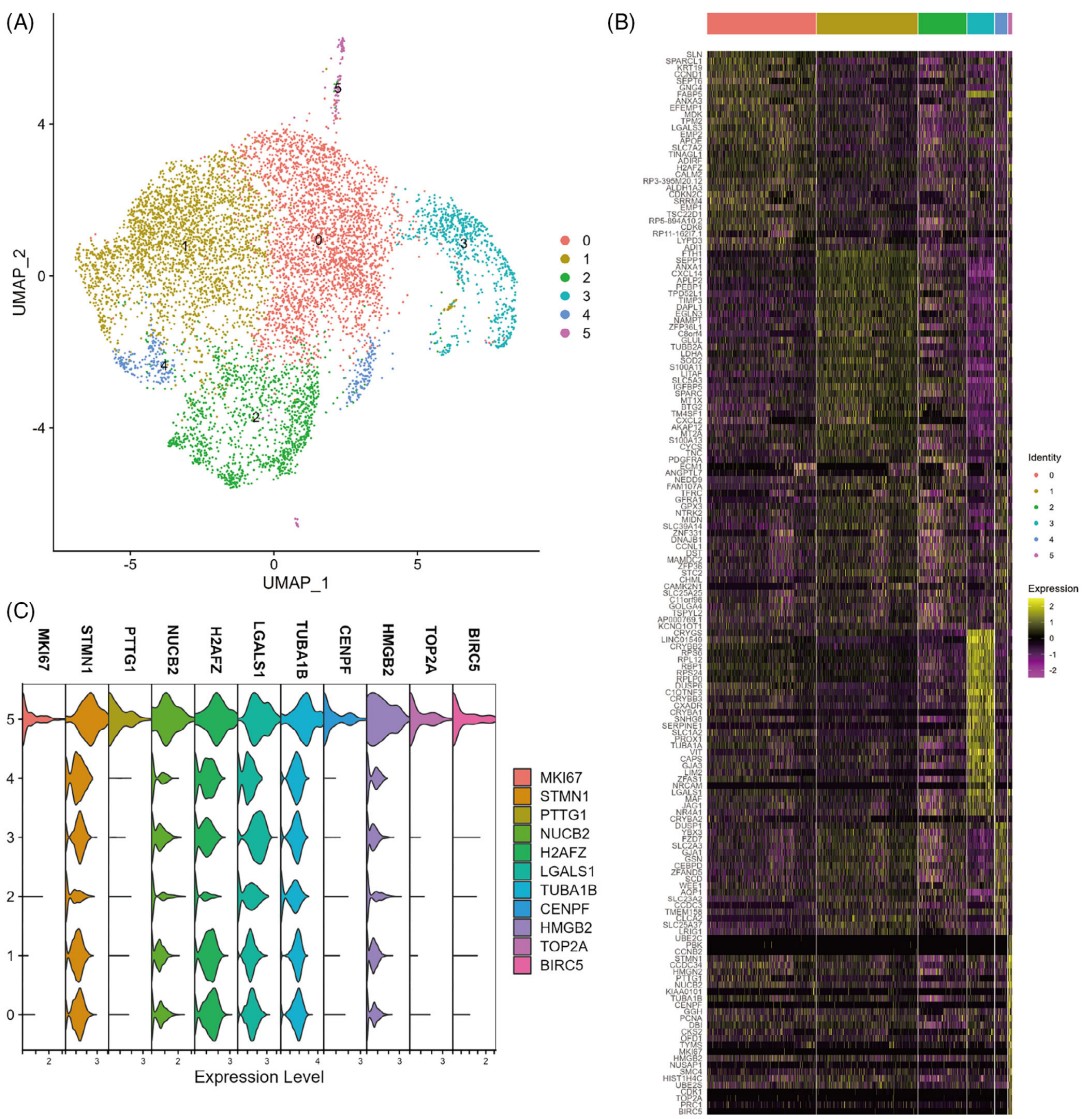
Identification of stem cells among ADAMTSL4^+^ cells. (A) uniform manifold approximation and projection (UMAP) of ADAMTSL4^+^ cells after reclustering. (B) Heat map of the top 30 differentially expressed genes in each cluster. (C) Violin plots of the expression of the top 10 marker genes in cluster 5 and MKI67 in the six clusters. (D) GO analysis of significantly differentially expressed genes in cluster 5.

Considering that the diffusion map suggested a common origin subpopulation of the two differentiation trajectories for ADAMTSL4^+^ cells, we extracted ADAMTSL4^+^ cells for reclustering (Figure 7A) and performed differential analysis of gene expression at a resolution of 0.2 (Figure 7B). We found that cluster 5 (104, 0.06%) showed a distinct stemness signature as indicated by expression of the cell proliferation marker MKI67, a gene commonly used for stem cell identification, and high expression of STMN1 relative to other clusters. Previous studies have shown a population of high STMN1‐expressing MIKI67^+^ cells in the isthmus that were characteristic of actively cycling stem cells.^25^ Additionally, PTTG1,^26^, ^27^ CENPF,^28^ TOP2A,^29^, ^30^ and BIRC5^31^ have been effectively studied as biomarkers for various types of stem cells (Figure 7C). Next, we performed GO functional enrichment analysis of the significantly differentially expressed genes in cluster 5, which showed that cluster 5 cells were closely related to the cell division process (Figure 6D). Taken together, cluster 5 may be lens epithelial stem/progenitor cells and exhibit the characteristics of STMN1^high^MKI67^+^PTTG1^+^CENPF^+^TOP2A^+^BIRC5^+^.

## 4 DISCUSSION

We used single‐cell RNA sequencing to comprehensively map the transcriptomes of 23,247 cells (D1–4) in lens superficial tissue. Our analysis identified two distinct subtypes of LECs and a new type of LFC in the superficial tissue of the human lens characterized by high expression of C8orf4, ADAMTSL4 and CD24, respectively. Moreover, we found a subpopulation of ADAMTSL4^+^ cells exhibiting STMN1^high^MKI67^+^PTTG1^+^CENPF^+^TOP2A^+^BIRC5^+^, which might be lens epithelial stem/progenitor cells. We also revealed two trajectories of LEC differentiation and changes in expression of some important genes during differentiation.

These cells are located in the different regions of the human lens. C8orf4^+^ cells were mainly in the anterior central region of the lens epithelium, and ADAMTSL4^+^ cells were mainly in the equator and pre‐equatorial region (Figure 3A). Interestingly, CD24 was expressed only in young elongating fibre cells directly adjacent to the epithelium around the entire lens, but not in the epithelium.

Our results of the cell clusters and their specific markers were different from the cell atlas of the human anterior segment reported by van Zyl et al., which classified lens cells into anterior epithelium, equatorial epithelium, transitional epithelium, early fibre and fibre. In our study, the selectively expressed markers of these five cell clusters did not show high expression (Figure S1C,D). Because the input material was cellular RNA rather than nuclear RNA in our study, it is not surprising that some of the most highly enriched gene sets were different from the results using snRNA‐seq samples, which might reflect the technical differences between the two sequencing technologies.

The ADAMTSL4 gene encodes an ADAMTS‐like protein involved in biogenesis the of fibrillin microfibrils. In mice, ADAMTSL4 is localized to the equator of the lens epithelium and plays an important role in stable anchorage of zonule fibres to the lens capsule.^6^ Mutations in ADAMTSL4 have been reported to cause ectopia lentis and ectopia lentis et pupillae. ADAMTSL4 is widely distributed in the normal human eye, especially the ciliary body and lens capsule in the equatorial region. Lens immunostaining showed that ADAMTSL4 localizes in the lens cortex in the equatorial region.^32^ The current study revealed that ADAMTSL4 was also expressed in LECs in equatorial and pre‐equatorial regions, and might be a selectively expressed marker of these cells. C8orf4, which is upregulated by various cellular stresses including UV irradiation and oxidative stress,^33^ is a upstream regulator of the Wnt/β‐catenin pathway.^34^ This pathway has a critical role in maintaining the LEC phenotype and delaying fibre cell differentiation.^35^, ^36^, ^37^ As a marker of cancer stem cells, CD24 is widely expressed in various cancer stem cells and various cells of the nervous system. It is also related to the cell adhesion function and immune cell differentiation, and is highly expressed in lymphoid progenitor cells. In the adult central nervous system, CD24 is limited to the secondary neurogenesis region.^38^, ^39^

Our study showed the epithelium in the central zone is important for the lens response to hypoxia and nutrient exchange (Figure 3B, Figure S4A–C). The microenvironment of central region cells in the lens epithelium limits access to nutrients, but its particular location requires that the central region epithelium maintains normal morphology and activity under limited oxygen levels and nutrients. The cellular response to low oxygen promotes cell survival and energy preservation.^40^

Epithelial cells in equatorial and pre‐equatorial regions are closely associated with cytoplasmic translation and lens development (Figure 3C, Figure S4D–F). There is substantial evidence for LECs in this region to be a population of cells with high proliferative activity that differentiate towards LFCs.^41^, ^42^, ^43^, ^44^ Therefore, it is plausible that they exhibit lens development‐related and active cytoplasmic translational functions. In terms of the possible location of lens epithelial stem/progenitor cells, they are thought to be located in the pre‐equatorial region of the lens epithelium,^43^, ^45^, ^46^, ^47^, ^48^ but their specific location and molecular characteristics remain to be clarified. Our results showed that epithelial cells in equatorial and pre‐equatorial regions specifically expressed ADAMTSL4 and exhibited high expression of stemness‐ and proliferation‐related genes such as ALDH1A3 and HES5. Thus, the subpopulation of ADAMTSL4^+^ cells that specifically expresses MKi67, BIRC5, PTTG1 and TOP2A may be lens epithelial stem/progenitor cells, and various cell states may exist in the transition of epithelial cells to fibre cells.

The neat alignment of lens fibres and growth control are important prerequisites to maintain lens transparency.^49^, ^50^, ^51^ Our results suggested that LECs express CD24 during differentiation into LFCs accompanied by cessation of cell proliferation and further progression of fibre differentiation as epithelial cells differentiate into fibre cells (Figure 3D, Figure S4G–I).^38^, ^39^ A study of the cornea reported that CD24 expression is associated with cellular homeostasis, the proliferation/differentiation balance of transit amplifying cells and the proliferation/maturation of directionally differentiated cells.^52^ Thus, CD24^+^ fibre cells might play a very important role in the maturation and orderly arrangement of lens fibres.

On the basis of our gene expression data, the LECs in the central region and the lens superficial fibre cells that express CD24 appear to have one common progenitor population of ADAMTSL4^+^ cells (Figures 5C and 6A). This population that expresses differentiation markers and progenitor markers gives rise to intermediate states of either the central epithelium or superficial fibre cells. We subsequently found this population of ADAMTSL4^+^ cells with various stemness features by reclustering analysis (Figure 7B,C), and GO functional enrichment analysis showed a close association with the biological process of cell division (Figure 7D). Therefore, this population of cells might be the lens epithelial stem/progenitor cells in a self‐renewal state. However, further research is needed for clarification. Furthermore, we characterized gene expression patterns along the different hierarchy and identified lineage‐specific genes, thereby enabling us to delineate the transcriptional events that regulate differentiation of LECs. Therefore, we suggest that the superficial fibre lineage represents the normal pathway through which LECs differentiate into LFCs, whereas the central epithelium lineage may represent a pathway to maintain LECs in the anterior central region of the human lens. Additionally, the unknown cluster is an important grouping, which might be a combination of several unique populations derived from three donors, reflecting the heterogeneity of the different ages (Figure S1A). However, because of the limited biological replicates available for age difference analysis, more samples and further study of this cluster are needed.

In summary, our data provide an unbiased view of LEC differentiation into fibre cells, a possible pathway to maintain central LECs, and a cluster of cells that might be lens epithelial stem/progenitor cells. This information supports some previously formed hypotheses in the lens field and describes differentiation processes at a high cellular resolution.

## AUTHOR CONTRIBUTIONS

Meng‐Chao Zhu performed the experiment and all the computational analyses and wrote the manuscript. Wei Hu, Yi Feng and Xiao‐Yu Tong performed the transparency experiment. Lei Lin collected the lens superficial cells. Jin Li, Xiu‐Feng Huang and Jian‐Zhong Su conceptualised the study. Qing‐Wen Yang, Lu Zhang, Jia‐Lin Xu, Yi‐Tong Xu performed the experiment. Jia‐Sheng Liu, Meng‐Di Zhang, Kai‐Yi Zhu, Ke Feng contributed the data acquisition and analyses.

## CONFLICT OF INETEREST STATEMENT

The authors declare no competing financial interests.

## Supporting information


**Figure S1.** Sample composition in single‐cell data. (A) UMAP of 21,711 single cells with three samples. (B) Proportion of different samples in the two epithelial cell subpopulations. (C) Expression of cell type markers of lens cells in our data in accordance with the cell atlas of the human ocular anterior segment. (D) Dot plot of genes selectively expressed by lens cells in accordance with the cell atlas of the

**Figure S2.** Difference in tissue clearance staining of the three different ages. (A) Detailed imaging of CD24 (red) in the 6M lens. (B, C) Spatial location and thickness of CD24 from the anterior capsule to the equator and posterior capsule in the three ages of lenses.

**Figure S3.** GO enrichment analysis of pseudotime‐dependent genes. GO analysis of superficial fibre lineage‐activated genes (A), repressed genes (B), transient genes (C), central epithelium lineageactivated genes (D), and repressed genes (E).

**Figure S4.** GO enrichment analysis of differentially expressed genes. Cnetplot of GO function enrichment analysis showing gene networks under enrichment pathways of C8orf4+ cells (A), ADAMTSL4+ cells (D), and CD24+ cells (G). Each dot represents a gene. The dot colour represents the expression level. Lines of different colours represent different enrichment pathways. The UpSet plot shows the number of overlapping genes between different enrichment pathways of C8orf4+ cells (B), ADAMTSL4+ cells (E), and CD24+ cells (H). Tree plot hierarchically clustered the terms of enrichment results from C8orf4+ cells (C), ADAMTSL4+ cells (F), and CD24+ cells (I).

Data S1.

Data S2.

Data S3.

## Data Availability

The authors declare that all data supporting the findings of this study are available in the article and its supplementary information files or from the corresponding author upon reasonable request. The RNA sequencing data are available online at https://github.com/tiger1916/lens-scRNA. All computational analyses were performed in R (version 4.1.1) using standard functions.
