# Supplementary figures and images for: Single‐cell RNA sequencing reveals new subtypes of lens superficial tissue in humans
Source: Cell Prolif. 2023 Apr 14;56(11):e13477. doi: 10.1111/cpr.13477 (PMC10623935; doi:10.1111/cpr.13477)

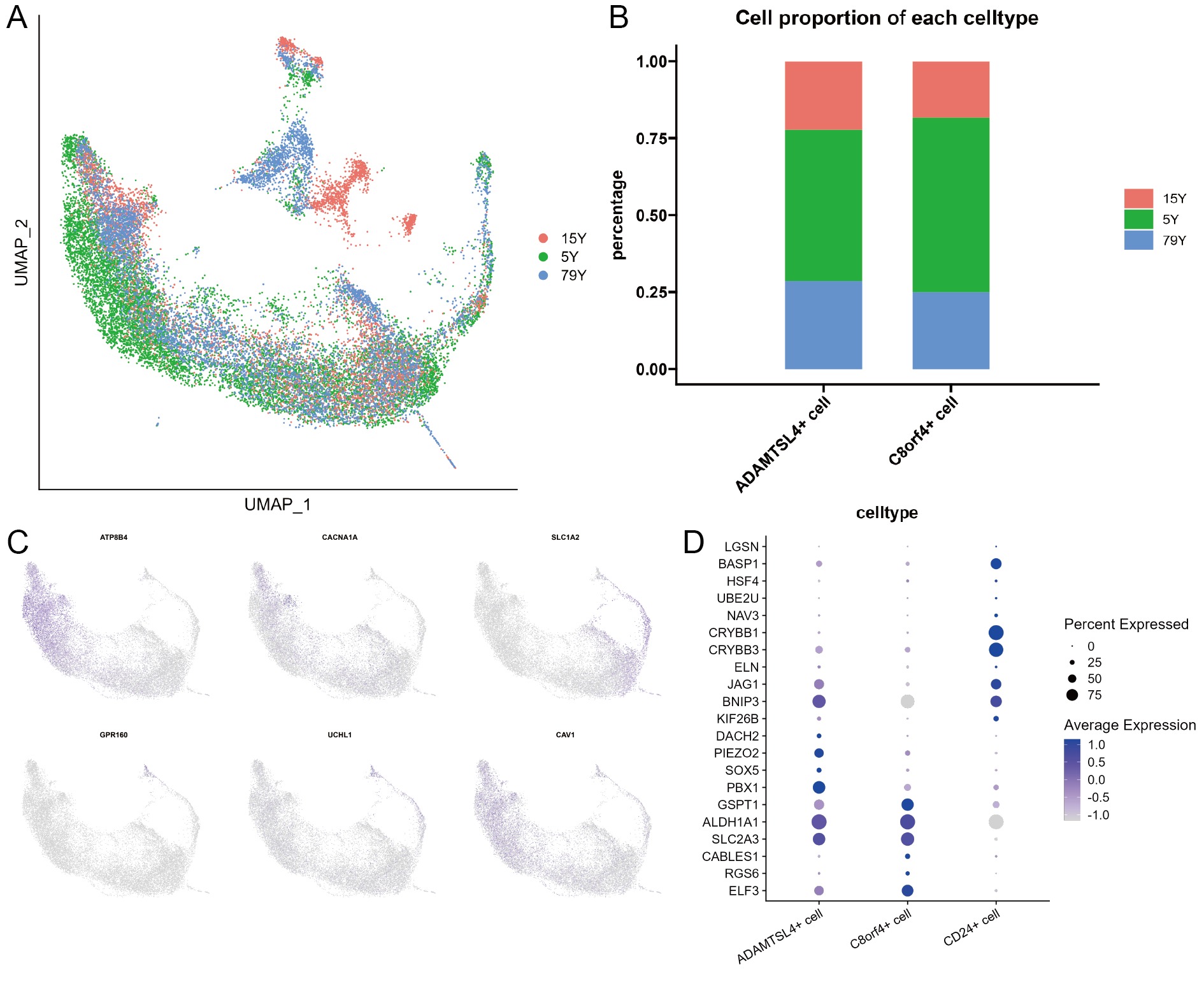

Supplement: Supplementary file 1 — Figure S1. Sample composition in single‐cell data. (A) UMAP of 21,711 single cells with three samples. (B) Proportion of different samples in the two epithelial cell subpopulations. (C) Expression of cell type markers of lens cells in our data in accordance with the cell atlas of the human ocular anterior segment. (D) Dot plot of genes selectively expressed by lens cells in accordance with the cell atlas of the [file CPR-56-e13477-s004.png]

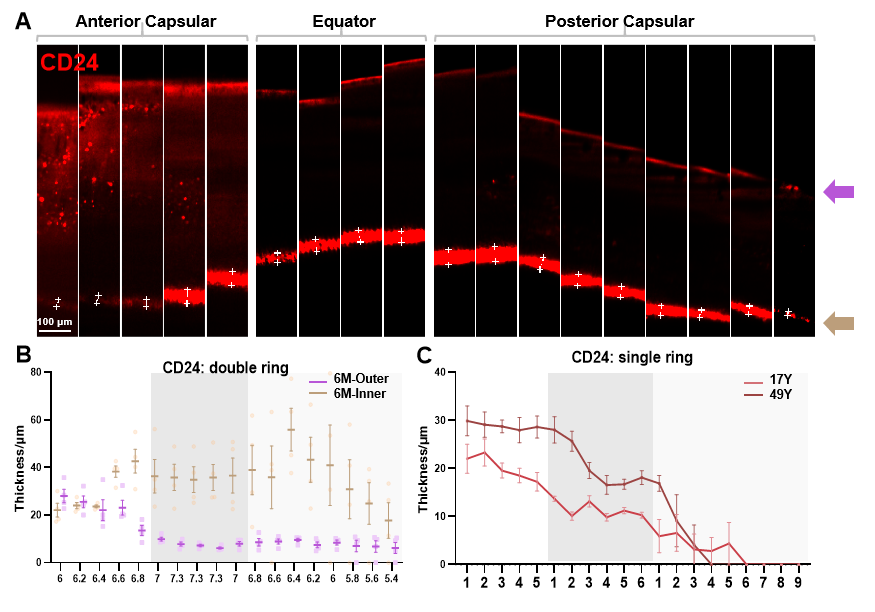

Supplement: Supplementary file 2 — Figure S2. Difference in tissue clearance staining of the three different ages. (A) Detailed imaging of CD24 (red) in the 6M lens. (B, C) Spatial location and thickness of CD24 from the anterior capsule to the equator and posterior capsule in the three ages of lenses. [file CPR-56-e13477-s005.png]

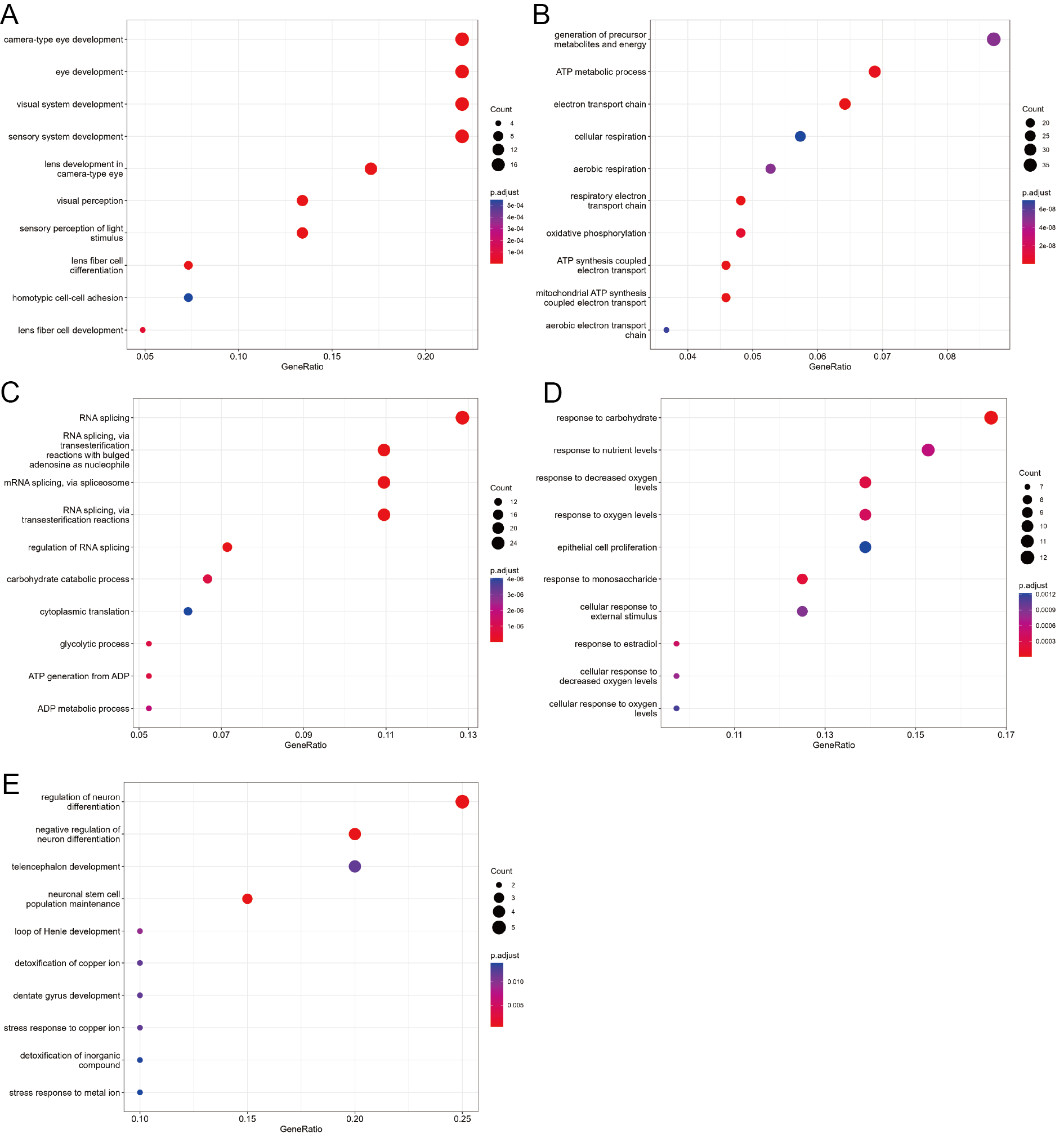

Supplement: Supplementary file 3 — Figure S3. GO enrichment analysis of pseudotime‐dependent genes. GO analysis of superficial fibre lineage‐activated genes (A), repressed genes (B), transient genes (C), central epithelium lineageactivated genes (D), and repressed genes (E). [file CPR-56-e13477-s001.png]

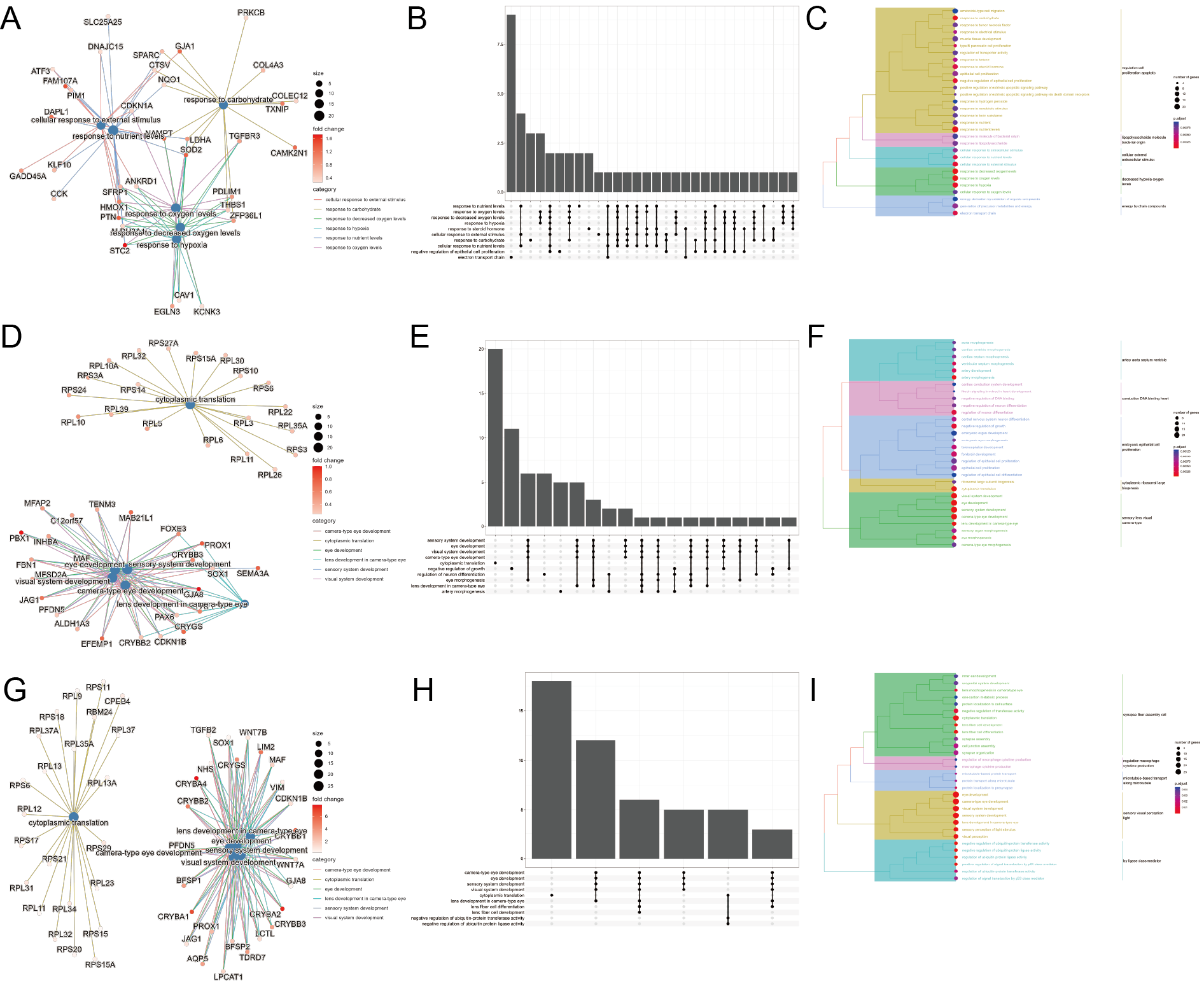

Supplement: Supplementary file 4 — Figure S4. GO enrichment analysis of differentially expressed genes. Cnetplot of GO function enrichment analysis showing gene networks under enrichment pathways of C8orf4+ cells (A), ADAMTSL4+ cells (D), and CD24+ cells (G). Each dot represents a gene. The dot colour represents the expression level. Lines of different colours represent different enrichment pathways. The UpSet plot shows the number of overlapping genes between different enrichment pathways of C8orf4+ cells (B), ADAMTSL4+ cells (E), and CD24+ cells (H). Tree plot hierarchically clustered the terms of enrichment results from C8orf4+ cells (C), ADAMTSL4+ cells (F), and CD24+ cells (I). [file CPR-56-e13477-s007.png]
